# Biochemical and Biophysical Characterisation of the Hepatitis E Virus Guanine-7-Methyltransferase

**DOI:** 10.3390/molecules27051505

**Published:** 2022-02-23

**Authors:** Preeti Hooda, Mohd Ishtikhar, Shweta Saraswat, Pooja Bhatia, Deepali Mishra, Aditya Trivedi, Rajkumar Kulandaisamy, Soumya Aggarwal, Manoj Munde, Nemat Ali, Abdullah F. AlAsmari, Mohd A. Rauf, Krishna K. Inampudi, Deepak Sehgal

**Affiliations:** 1 Virology Lab, Department of Life Sciences, Shiv Nadar University, Greater Noida 201314, India; ph236@snu.edu.in (P.H.); iftikharbiochem@gmail.com (M.I.); shweta.saraswat@nii.ac.in (S.S.); pb671@snu.edu.in (P.B.); mishratani2012@gmail.com (D.M.); at662@snu.edu.in (A.T.); 2 Department of Biophysics, All India Institute of Medical Sciences (AIIMS), New Delhi 110029, India; danielrajkumar@aiims.ac.in; 3 School of Physical Sciences, Jawaharlal Nehru University (JNU), New Delhi 110067, India; soumya22_sps@jnu.ac.in (S.A.); mmunde@mail.jnu.ac.in (M.M.); 4 Department of Pharmacology and Toxicology, College of Pharmacy, King Saud University, P.O. Box 55760, Riyadh 11451, Saudi Arabia; nali1@ksu.edu.sa (N.A.); afalasmari@ksu.edu.sa (A.F.A.); 5 Department of Pharmaceutical Sciences, Wayne State University, Detroit, MI 48201, USA; ahmarrauf2@gmail.com

**Keywords:** hepatitis E virus, methyltransferase, fluorescence quenching, protein–ligand interaction, protein stability, enzyme assay

## Abstract

Hepatitis E virus (HEV) is an understudied pathogen that causes infection through fecal contaminated drinking water and is prominently found in South Asian countries. The virus affects ~20 million people annually, leading to ~60,000 infections per year. The positive-stranded RNA genome of the HEV genotype 1 has four conserved open reading frames (ORFs), of which ORF1 encodes a polyprotein of 180 kDa in size, which is processed into four non-structural enzymes: methyltransferase (MTase), papain-like cysteine protease, RNA-dependent RNA polymerase, and RNA helicase. MTase is known to methylate guanosine triphosphate at the 5′-end of viral RNA, thereby preventing its degradation by host nucleases. In the present study, we cloned, expressed, and purified MTase spanning 33–353 amino acids of HEV genotype 1. The activity of the purified enzyme and the conformational changes were established through biochemical and biophysical studies. The binding affinity of MTase with magnesium ions (Mg^2+^) was studied by isothermal calorimetry (ITC), microscale thermophoresis (MST), far-UV CD analysis and, fluorescence quenching. In summary, a short stretch of nucleotides has been cloned, coding for the HEV MTase of 37 kDa, which binds Mg^2+^ and modulate its activity. The chelation of magnesium reversed the changes, confirming its role in enzyme activity.

## 1. Introduction

Hepatitis E virus (HEV) is inherently hepatotropic, causing acute hepatitis and chronic infection in immunocompromised patients, as well as leading to extrahepatic manifestations in some patients [1,2,3,4]. Globally, hepatitis E accounts for an estimated mortality rate of ~3.3% in the infected population and causes fulminant hepatitis failure in 25–30% of infected pregnant women [5]. HEV has a genome of ~7.2 kb plus-stranded RNA with a 5′-methylguanine (m^7^G) cap accorded by guanylyltransferase (GTase) and methyltransferase (MTase). The RNA capping is essential for the viruses to evade the host immune system and produce other viral proteins by protecting the viral mRNA from nucleases. In the case of HEV, the 5′ m^7^G cap has been demonstrated to play an additional role, increasing infectivity in non-human primates and cultured hepatoma cells [6,7]. Despite being an important enzyme, few studies have examined the functional and structural aspects of the HEV MTase. Magden et al. 2001 expressed a 110 kDa protein from the 1–979 amino acid region of HEV cDNA and demonstrated both the activities of MTase and GTase [8]. Before that, using computational homology modelling, Koonin et al. derived that the MTase domain lay in the region of aa 56–240 of the HEV genome [9]. Through bioinformatic studies, Emerson et al. predicted that the MTase lay in the region of aa 33–353 of HEV genotype 1 [6]. This fragment was further preferred since it formed a section of the MTase domain of aa 1–979 expressed by Magden and overlapping the region of amino acids 56–240, computationally predicted to be MTase by Koonin. Howver, expressing the protein containing this predicted fragment and validating the MTase activity is an unexplored area.

Other viruses in which capping is essential for MTase activity include the Dengue virus [10], coronaviruses [11], and flaviviruses [12]. MTase is an integral enzyme required for capping, which is dependent on magnesium (Mg^2+^) as a cofactor for its activity [13]. The dependence of MTase on divalent cations for its activity has been observed in the case of many viruses, such as the Zika virus [14] and respiratory syncytial virus (RSV) [15]. Many viruses, such as the Chlorella virus and hepatitis C virus (HCV), coronaviruses, and flaviviruses, also need Mg^2+^ for the activity of viral enzymes [16,17,18,19,20]. Alteration of the activity may be due to conformational changes resulting from the binding of magnesium to the enzyme. Following this hypothesis, the role of Mg^2+^ in the MTase activity of coronaviruses has been attributed to the conformational changes in the nsp10/nsp16 enzyme complex for 2′O-methylation [21]. The binding is postulated to induce the change in the structural, conformational, and interactional properties of MTase. As seen in the Dengue virus, Mg^2+^ stabilises the RNA cap by coordinating with the inverted triphosphate moiety from the solvent-exposed side of the RNA cap [22].

Briefly, in the present study, we have reported and expressed the HEV MTase from the predicted domain of the 33–353 amino acid fragment of HEV genotype 1. The molecular weight of the enzyme with MTase activity was found to be 37 kDa. The enzyme activity was shown to be associated with Mg^2+^ using biochemical and biophysical studies.

Our work suggested that HEV MTase requires Mg^2+^ for its activity, and future studies should help to establish the direct relationship between RNA capping and host cellular metal ions. Further, the identification of drug-like inhibitors that are structurally compatible with the domains responsible for the enzyme activity could be performed. Using this information, the co-crystallisation of MTase with magnesium can be performed, to elucidate the exact binding pocket of Mg^2+^ and hence design structurally compatible inhibitors.

## 2. Results

### 2.1. Expression and Purification of MTase

**Figure 1:**
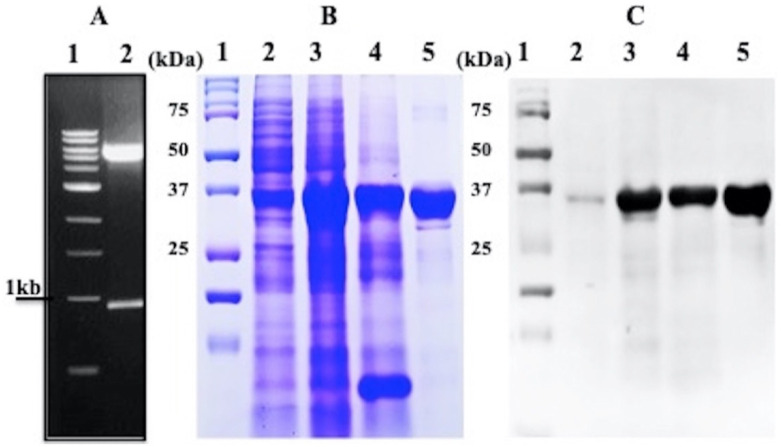
**Cloning, expression, and purification of MTase:** (**A**) The cloned MTase gene (969 bp) was confirmed by restriction digestion using NdeI and XhoI (Panel A). (**B**) Coomassie-stained gel representing the expressed and purified fractions of HEV MTase. Lane 1, marker; lane 2, uninduced cell lysate; lane 3, induced cell lysate; lane 4, solubilised fraction; lane 5, Ni-NTA purified fraction. (**C**) Western blotting analysis using anti-HEV MTase primary antibody. Lane 1, marker; lane 2, uninduced cell lysate; lane 3, induced cell lysate; lane 4, solubilised fraction; lane 5, Ni-NTA purified fraction.

For studying the biochemical and biophysical characteristics of the enzyme MTase, the gene for MTase was cloned into the pET-28a vector and validated by restriction digestion of a ~969 bp fragment (Figure 1A) and confirmed by DNA sequencing (data not shown). The recombinant MTase construct was transformed into BL21-DE3 cells to express the protein, as demonstrated using SDS-PAGE and Western blotting with proper controls (Figure 1B,C; Lanes 2 and 3). The protein was solubilized using different detergents, but achieved maximum solubility with 0.5% NLS (Figure 1B,C; Lane 4). The recombinant protein of 37 kDa, with a His-tag, was purified using Ni-NTA chromatography and confirmed to be MTase, as shown by SDS-PAGE and western blotting using epitope-specific antibodies (Figure 1B,C; Lane 5). In conclusion, the protein was purified and confirmed to be MTase through enzyme assay using GTP as the substrate.

### 2.2. MTase Activity of 37 kDa Protein

#### 2.2.1. Enzyme Kinetics

**Figure 2:**
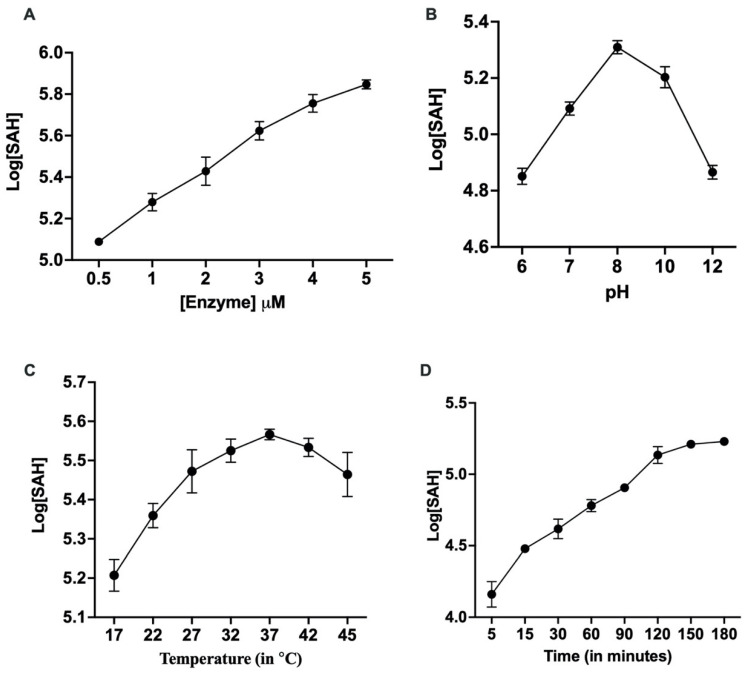
**Effect of various parameters on MTase activity:** The MTase activity was optimised by adjusting various parameters. (**A**) MTase activity at varying enzyme concentrations. (**B**) Enzyme activity at different pH values. (**C**) Enzyme activity at different temperatures. (**D**) Enzyme activity at various time points. The observations are made from experiments performed in triplicate. Each data point on the graph represents the mean value, and the error bars indicate the standard deviation.

MTase kinetics were studied as described in Materials and Methods to determine the nature of the enzyme and its activity. The activity of the purified protein was determined through different parameters, including various ranges of temperature, pH, and time. A linear increase in the enzyme activity was observed upon the increase in MTase concentration from 0–5 μM (Figure 2A). Further, enzyme activity was studied in the pH range from 6 to 12, leading to a bell-shaped curve with an optimal pH of 8.0 (Figure 2B). Additionally, the optimal temperature of the enzyme activity was found to be 37 °C (Figure 2C), above which the enzyme became denatured. Further, through the time-course assay, the optimal enzyme activity was found at 150 min, after which it plateaued (Figure 2D). For all future studies, the concentration of the enzyme used was 0.675 μM, while the concentration of GTP used was 0.4 mM. The graphs were plotted in terms of S-adenosyl homocysteine (SAH) concentration, determined using the standard curve. All the reactions were performed in triplicate, and the readings were subtracted from those of the no enzyme control. Each data point in the graph represents the mean value, and the error bars indicate the standard deviation.

#### 2.2.2. MTase Activity in the Presence of Guanosine Triphosphate (GTP) as a Substrate

**Figure 3:**
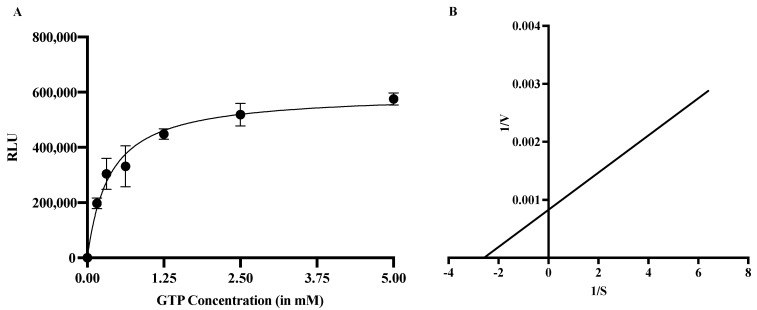
**Kinetic studies of MTase:** (**A**) The reaction mixture contained 1 µM SAM and different concentrations of GTP. Each data point represents the mean value, and the error bars indicate the standard deviation. The K_m_ value of the substrate, GTP, was calculated using the Michaelis–Menten equation and found to be approximately 0.38 mM. (**B**) The Lineweaver–Burke plot was produced by GraphPad Prism 9. A straight-line equation was determined using K_m_ and V_max_ (Y = 0.0003207 × X + 0.0008274). The calculated K_m_ and V_max_ values using the Lineweaver–Burke plot were 0.387 mM and 120,496, respectively.

The MTase kinetics were performed in the presence of GTP as its substrate while keeping the concentrations of S-adenosyl methionine (SAM) (methyl donor) and the enzyme constant at 1 μM and 0.675 μM, respectively. The concentration of the GTP substrate (methyl acceptor) ranged from 0 to 10 mM. The Michaelis–Menten equation was used to calculate the k_m_ value for GTP, which was found to be 0.387 mM (Figure 3A). The k_m_ value was also determined using a Lineweaver–Burke plot produced by GraphPad Prism 9. The straight-line equation for the plot, Y = 0.0003207 × X + 0.0008274, was used to calculate the k_m_ and V_max_ values (Figure 3B). The calculated k_m_ and V_max_ values from the Lineweaver–Burke plot were found to be 0.387 mM and 120,496, respectively.

#### 2.2.3. Substrate Specificity of MTase

**Figure 4:**
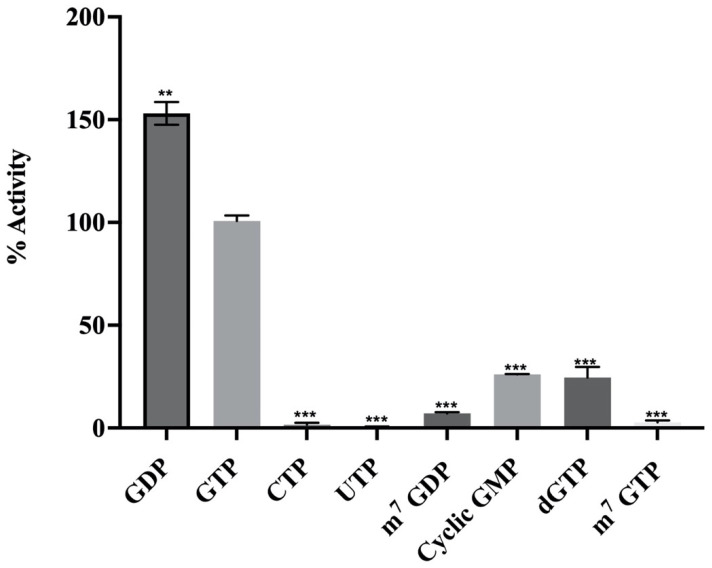
**Effect of different substrates on HEV MTase activity:** The graph represents the activity of MTase using different substrate analogues. The bars represent various nucleotide analogues, as indicated in the figure. The values have been normalised to the percentage activity of the enzyme in the presence of GTP. The enzyme assay was performed in triplicate; the graph represents the mean value while the error bar indicates the standard deviation. The statistical significance of the data was determined using Student’s *t*-test. ** *p*-value < 0.05, *** *p*-value < 0.0005.

The substrate specificity of the enzyme was studied using different nucleotides, cytidine triphosphate (CTP), uridine triphosphate (UTP), cyclic adenosine monophosphate (cAMP), guanosine diphosphate (GDP), GTP, and deoxy guanosine triphosphate (dGTP), as well as the capped analogues, 7-methyl guanosine diphosphate (m^7^-GDP) and 7-methyl-guanosine triphosphate (m^7^-GTP). The reaction was performed, in which 0.675 μM of MTase was tested for enzyme activity in the presence of 0.5 mM of different nucleotide triphosphates (NTPs) as cap analogues, as mentioned above. The concentration of SAM, the methyl donor, was kept constant at 1 μM in the reactions. The activity assay demonstrated that GDP is a better substrate than GTP, while minimal activity was observed for CTP, UTP, cAMP, and dGTP (Figure 4). The cap analogues, m^7^-GDP and m^7^-GTP, showed minimal activity. These results showed the specificity of MTase activity, indicating that there must be a free position on the substrate to transfer the methyl group. The minimal activity for m^7^-GDP and m^7^-GTP was observed since the N-7 position of the guanosine is already methylated [8] (Figure 4).

#### 2.2.4. Effect of Magnesium (Mg^2+^) on MTase Activity

**Figure 5:**
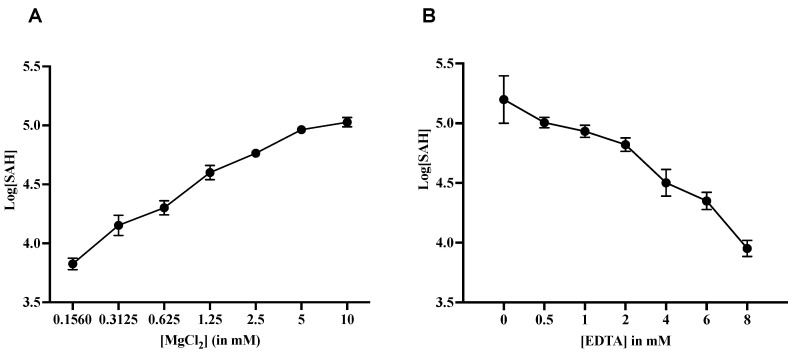
**Effect of magnesium on MTase activity**: (**A**) The graph represents MTase activity when increasing the concentration of MgCl_2_ up to 10 mM. (**B**) The effect of EDTA on the enzyme activity. The graphs represent the mean value of three different readings and the error bars indicate the standard deviation.

Although divalent cations are crucial for the activity of many viral enzymes, no study has been observed the role of divalent cations on MTase activity. Hence, MTase activity in response to varying concentrations of Mg^2+^ was studied. This resulted in the linear increase in MTase activity for up to 2 mM of magnesium, after which it became saturated (Figure 5A). The effect of another important cation, Ca^2+^, was checked for its activity of the enzyme, but no effect on MTase activity was seen (data not shown). To further validate the impact of Mg^2+^, the reaction was performed in the presence of EDTA, which is supposed to be a chelating agent for the magnesium. When increasing the concentration of EDTA from 0 to 8 mM, the enzyme activity decreased significantly, establishing that the presence of magnesium is essential for the MTase activity (Figure 5B).

### 2.3. Circular Dichroism (CD) Analysis

**Figure 6:**
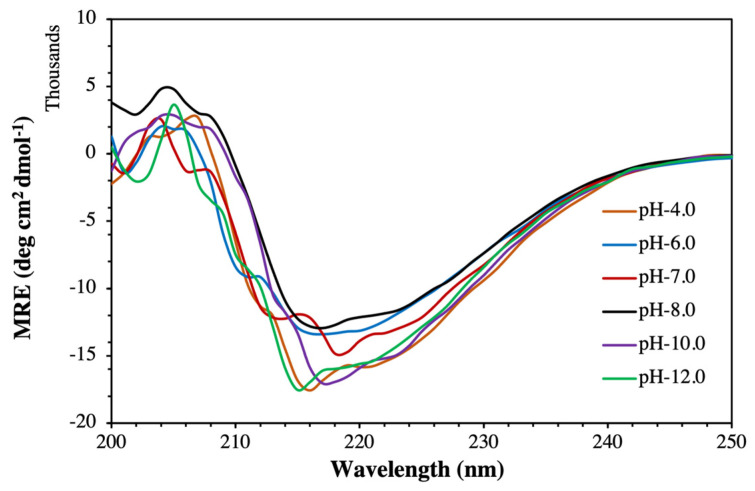
**Far-UV CD spectra of MTase:** To determine the change in the secondary structure of MTase in the presence of N-lauryl sarcosine sodium salt and NaCl at pH 4.0, 6.0, 7.0, 8.0, 10.0, and 12.0 at 25 °C.

Far-UV CD experiments were performed to investigate the changes in secondary structural content of MTase at different pH values or Mg^2+^ concentrations [23]. The obtained MTase spectra showed two negative peaks around 208 nm and 222 nm. A positive peak at 195 nm (not shown in the figure due to high HT values at this region) reflects a characteristic α-helical spectrum of MTase (Figure 6). In addition, the negative peak at 218 indicates the presence of the β-sheets. Overall, at pH 8.0, it contains 27% α-helix, 23% β-sheet, and the rest of the content as random coils, loops, turns, etc. Hence, we report that 50% of the MTase protein is comprised of α-helical and β-sheet structures. Similar percentage of α-helices and β-sheets for MTase were obtained by secondary structure prediction methods (data not shown). The secondary structure of MTase was calculated using online DichroWeb software (http://dichroweb.cryst.bbk.ac.uk/html/home.shtml) (accessed on 27 December 2021) based on the K2D model and analysed by the method of Chen et al. [24].

### 2.4. Structural and Thermal Stability of MTase in the Presence of Mg^2+^

**Figure 7:**
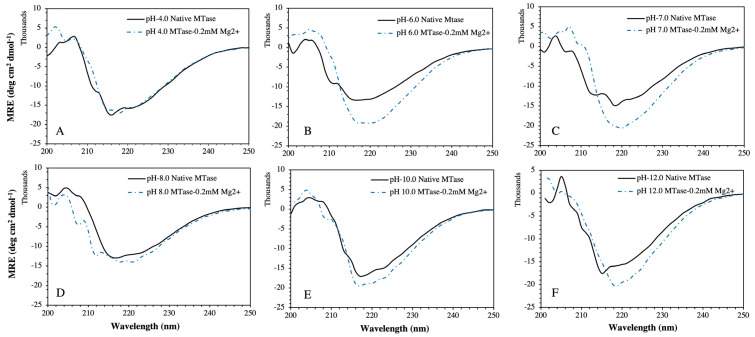
**Far-UV CD spectra of MTase:** CD spectra at (**A**) pH 4.0, (**B**) pH 6.0, (**C**) pH 7.0, (**D**) pH 8.0, (**E**) pH 10.0, and (**F**) pH 12.0 under standard temperature in the absence and presence of 0.2 mM Mg^2+^; all respective buffer solutions contain N-lauryl sarcosine sodium salt and NaCl to provide structural stability at respective pH.

The structural stability of MTase was investigated in the presence of Mg^2+^ at different pH conditions. The experimental results revealed a minimum change in the secondary structure of MTase in the presence of Mg^2+^ at pH 4.0 and pH 8.0 compared to pH 6.0, pH 7.0, pH 10.0, and pH 12.0 (Figure 7). As per our observations, Mg^2+^ is responsible for the change in the secondary structure at different pH conditions. However, it has been found that shifting the pH values towards acidic (pH 4.0) or alkaline (pH 12.0) is responsible for the increment in the α-helix content of MTase. This observation confirmed that the MTase secondary structure in the presence of 0.2 mM Mg^2+^ is more stable at pH 4.0 and pH 8.0 compared to other pH values. Therefore, we selected pH 8.0 for further studies, which is closer to physiological pH. This will help provide a comparative analysis for protein activity and drug discovery.

**Figure 8:**
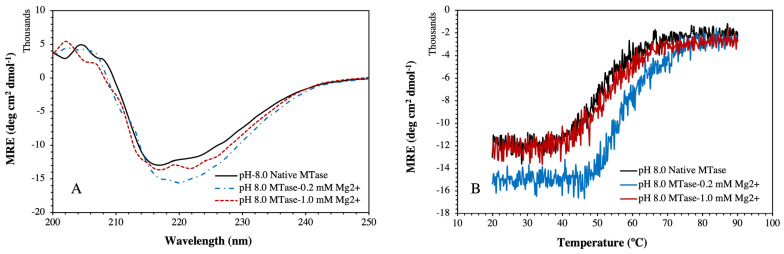
**Far-UV CD spectra and thermal denaturation of MTase:** (**A**) Far-UV Cd spectra at standard temperature (25 °C). (**B**) Thermal denaturation spectra of MTase at 222 nm in absence and presence of 0.2 mM and 1.0 mM Mg^2+^. Tris buffer of pH 8.0 containing N-lauryl sarcosine sodium salt and NaCl was used in each experimental sample under minimum concentrations.

Further, we have determined the effect of Mg^2+^ on MTase secondary structural changes by varying the Mg^2+^ concentrations (Figure 8A). We found that 0.2 mM Mg^2+^ effectively changed the secondary structure of MTase compared to 1.0 mM of Mg^2+^. Furthermore, the far-UV CD spectra revealed that lower Mg^2+^ concentrations induced the formation of β-sheets that was proportionally not supported at higher concentrations. Moreover, we determined the thermal stability at similar conditions. The obtained experimental denaturation graphs (Figure 8B) at 0.2 mM Mg^2+^ showed that the metal ions effectively induced the thermal stability of MTase compared to 1.0 mM (Table 1). Far UV-CD spectra and enzymatic assays also supported these results.

We calculated the thermal unfolding of MTase by the two-state folding–unfolding model and the related Equations (2) and (3), which are used to resolve the temperature mid-point (T_m_) by fitting the ellipticity. The results showed that secondary structural contents of MTase were transformed during the thermal denaturation process. The loss of protein function and structure were directly related to the decrease in the ellipticity at 222 nm with temperature. The obtained T_m_ values of native MTase were calculated at around ~58.8 °C, which changed significantly compared to the presence of Mg^2+^ (Figure 8B, Table 1). The thermal denaturation of MTase and waning of hydrophobic and other non-covalent interactions might be responsible.

### 2.5. Determination of Binding Affinity and Mechanism of Mg^2+^ Ion with MTase by Fluorescence Quenching Method

**Figure 9:**
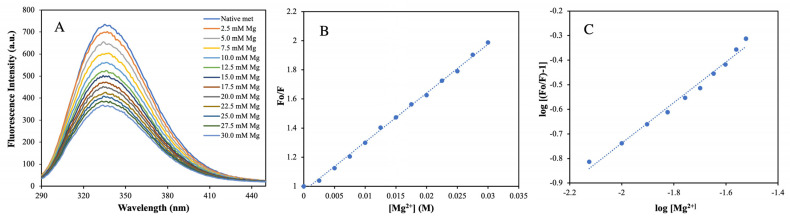
(**A**) Fluorescence quenching measurement: fluorescence emission spectra of MTase (5 µM) in the presence of Mg^2+^. (**B**) Stern–Volmer plot for the MTase–Mg^2+^ interaction. (**C**) Binding parameter measurements: plot of log [(Fo/F) − 1] vs. log [Mg^2+^] for the determination of binding constants and binding stoichiometry for the MTase–Mg^2+^ interaction at room temperature and pH 8.0.

We also performed fluorescence quenching experiments to determine the binding affinity of Mg^2+^ with MTase. For this, we titrated Mg^2+^ against MTase (5 µM) at 25 °C. Figure 9A shows that MTase possesses a sharp fluorescence emission peak around 340 nm when excited at 280 nm. The quenching of MTase fluorescence occurs by increasing Mg^2+^ to its saturation level, at a millimolar concentration. It is possible that Mg^2+^ intercalates with MTase at a site close to tryptophan or other aromatic amino acid residues. This region is predominantly responsible for the change in the emission peak at around 340 nm after excitation at 280 nm [25]. Therefore, an ongoing reduction in the emission spectral intensity of MTase has been found, without remarkable variation in the wavelength of maximal fluorescence emission (λ_max_) until the final quenching concentration [26].

To determine the binding affinity of MTase, we performed the titration of Mg^2+^ against MTase at 25 °C. However, the *k*_q_ value for the MTase–Mg^2+^ system was ten times higher than the highest scatter collision quenching constant of innumerable quenchers with polymers (2 × 10^10^ M^−1^s^−1^) [27]. This reflects that quenching is not commenced by dynamic diffusion, but arises by creating powerful complex formation between MTase and Mg^2+^. 

The intrinsic fluorescence intensity (FI) of aromatic amino acids decreases continuously by increasing the metal ion concentration. For example, the emission spectra become saturated at 30 mM Mg^2+^, as shown in Figure 9A. The decrease in FI upon adding ions was analysed using the Stern–Volmer equation. It is a fact that the slopes in Figure 9B indicate that the binding of the ligand to the protein is responsible for quenching [28]. Based on the Stern–Volmer plot, the *K*_q_ value of Mg^2+^ is 5.81 × 10^9^, reflecting lower scatter collision quenching value initiated by the dynamic diffusion of molecules.

The binding constant and related binding stoichiometry of Mg^2+^ were calculated as per log [(Fo/F) − 1] plotted against log [Mg^2+^], as presented in Figure 9C. The slope of these plots reveals that binding stoichiometry (*n*) and corresponding intercept value give the information about binding constant (*K*_b_), which was calculated from Equation (5), with computed values are reflected in Table 2. 

Therefore, our CD and fluorescence data imply that under slightly basic conditions (at pH 8.0), the structural and conformational alteration in MTase was minimal compared to other pH and more feasible for the study of interaction with other inhibitors in the presence of Mg^2+^. These consequences occur because of the pH-induced alteration in the vicinity of the metal-binding site of the MTase that is responsible for the change in the mode and mechanism of protein, and finally, the interaction of MTase with Mg^2+^ [29].

### 2.6. Isothermal Titration Calorimetry (ITC) Analysis

**Figure 10:**
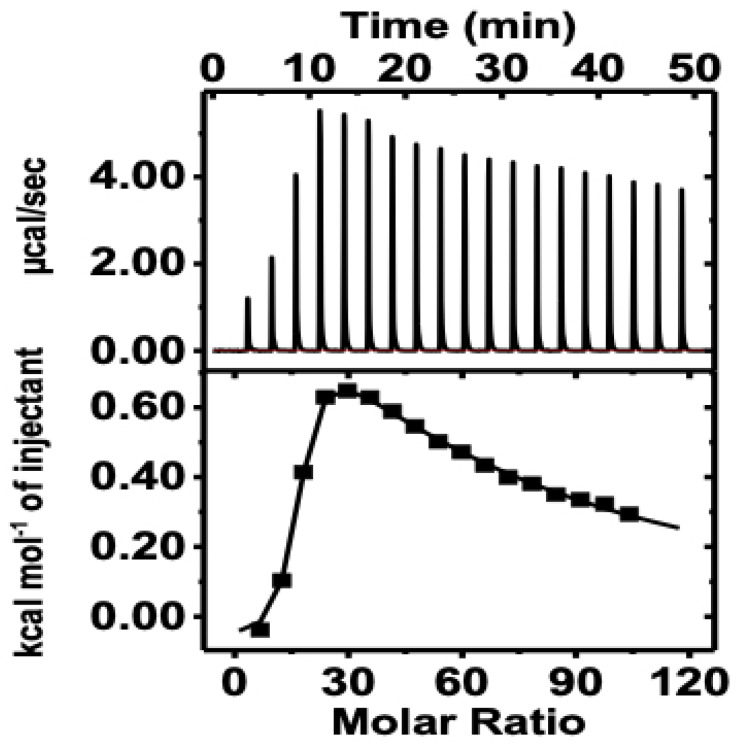
**ITC thermogram:** ITC thermogram showing the binding of protein with Mg^2+^. The experiment was performed at 25 °C; top panel, detected heat signals; bottom panel, integrated heat of reaction for each titration.

The thermodynamic parameters of Mg^2+^ upon binding to MTase were studied by ITC. The injected heat signals for Mg^2+^ binding with MTase and the integrated heat of the reaction for each injection are displayed in Figure 10. The calculated output results of the ITC data are shown in Table 3, which demonstrate that there are two binding sites available for Mg^2+^ in MTase. The first binding (6.7 × 10^4^ M^−1^) site is much stronger, which is driven by a small negative enthalpy (−0.08 ± 0.06 kcal/mol) and a large positive entropy (6.49 ± 0.10 kcal/mol). The favourable entropy contributes to the solvation entropy because of the loss of water from the binding interface, indicating the presence of electrostatic interaction during the complex formation. Meanwhile, the secondary binding site is weaker (3.0 × 10^2^ M^−1^) and endothermic. Based on the thermodynamic analysis, it is suggested that the protein–Mg^2+^ complexation is mainly driven by electrostatic interaction.

### 2.7. Binding Study of Magnesium with MTase 

**Figure 11:**
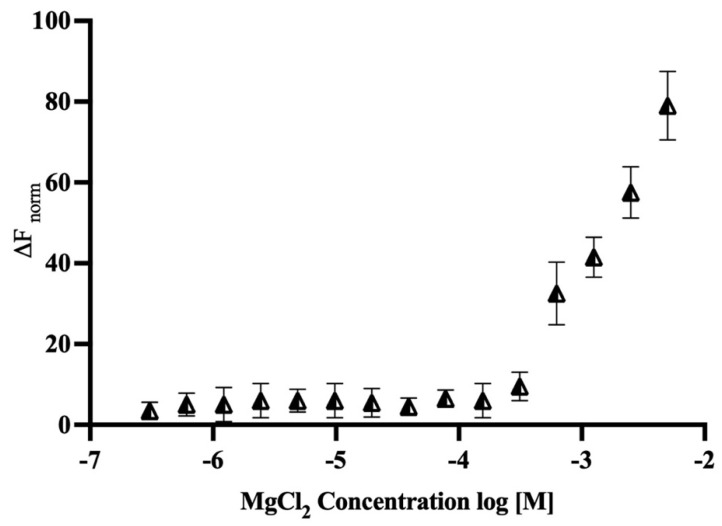
**Microscale thermophoresis:** Dose–response curve for the binding interaction between RED-Tris-NTA-labelled MTase and Mg^2+^. The concentration of RED-Tris-NTA-labelled MTase is constant, while the concentration of Mg^2+^ varies between 5 mM and 300 nM. The K_D_ value for MgCl_2_ with MTase is 15 μM. The *Y*-axis on the graph represents the fluorescence change, and the *X*-axis on the graph represents the concentration of Mg^2+^. The graph describes the values of three different experiments.

Further, the binding affinity of magnesium and MTase was determined using microscale thermophoresis (MST), a tool to study the interaction of the biomolecules. During the study, the MTase concentration was set at 50 nM, and the concentration of Mg^2+^ was varied from 5 mM to 300 nM in 16 different dilutions. The graph was plotted using the concentration of Mg^2+^ on the *X*-axis in log [M], while the *Y*-axis displayed the normalised fluorescence. The sample fluorescence is recorded during an MST experiment, starting with 3 s at ambient temperature to monitor steady-state fluorescence, followed by IR laser activation for a defined MST-on time. The MST analysis was performed using MO Control and MO Affinity Analysis Software (Monolith NT.115, NanoTemper Technologies, München, Germany). The calculated dissociation constant (K_D_) between MTase and Mg^2+^ is approximately 15 μM (Figure 11).

## 3. Discussion

The plus-stranded viruses have a 5′-capped genome catalysed by MTase [30,31] and found to be essential for their infectivity and replication [6,7]. In line with this, the 5′ non-coding region (NCR) of HEV RNA has been demonstrated to have an m^7^G-cap that is indispensable for its life cycle [6,32]. Similar results have been reported in alphavirus nsP1, tobacco mosaic virus P126, brome mosaic virus replicase protein 1a, and bamboo mosaic virus nonstructural protein [8]. Despite being an important enzyme that may act as a drug target, not many structural or functional studies have been conducted on MTase. Previously, the molecular weight of the active enzyme has been demonstrated to be 110 kDa, encoding amino acids 1 to 979 of the HEV genome [8]. In another study, using a computational approach, the MTase region has been predicted to be from 56 to 240 amino acids [9]. Emerson et al.’s computational predictions revealed that a region of 33–353 amino acids on the HEV pSK-HEV2 genome could exhibit MTase activity [6]. Therefore, we expressed this region to translate a protein of 37 kDa in size, as confirmed by western blotting and MALDI-TOF (Supplementary data). A recent study also demonstrated that a ~37 kDa MTase enzyme was processed from the HEV-ORF1 polyprotein when Huh7 cells were transduced with BacMam-HEV [33]. In our previous study, the digestion of ORF1 polyprotein, using cysteine protease, yielded a ~37 kDa protein, detected by MTase epitope-specific antibodies [34]. The enzyme was thus expressed and found to be active, as determined using the luminescence-based assay. The activity was altered by various cap analogues, as seen in an earlier study by Magden et al. [8]. The enzyme activity was also confirmed using enzyme kinetics and binding studies.

Another objective of this study is the metal dependency of MTase activity and several viral MTases, as other enzymes use divalent cations for their activity [17,18,19,20], which prompted us to study the effect of Ca^2+^ and Mg^2+^ on MTase activity. While there was no considerable effect on the enzyme activity was observed in the presence of Ca^2+^ (data not shown), significant MTase activity was observed in the case of Mg^2+^. The results confirmed that activity was decreased when Mg^2+^ was depleted by the chelating agent, EDTA. In eukaryotes, mRNA capping also requires Mg^2+^ for the catalysis of lysine–GMP intermediate formation [35]. The metal ions are known to stabilise the random coil regions of enzymes by forming metal-binding pockets and protecting the unstructured part from the protease activity; they act as electron donors at the catalytic centre to expand the biochemical palette and regulate a wide range of functions. 

In this work, we performed biophysical studies to explore the importance of Mg^2+^, which affects the stability of MTase at different ranges of pH and temperature. The far-UV-CD spectra (190–250 nm) clearly showed that MTase contains both α-helices (27%), and β-sheets (23%), but different pH environments induced changes in the secondary structure components. The similar secondary structural components were seen in most SAM-dependent MTases with a Rossmann-like fold. Further, the CD experiments at different Mg^2+^ concentrations revealed that the secondary structure was proportional to Mg^2+^ concentration, which is evident in metal-binding proteins. Thermal denaturation experiments in the presence of Mg^2+^ were also performed to understand the effect of metal ions on MTase folding by increasing the temperature. Analysis of the plot revealed the reduction in the percentage of the secondary structure with increased temperature, indicating the absence of any intermediate unfolding states during the thermal unfolding pathway of MTase. The obtained T_m_ values of MTase were calculated using native conditions that significantly increased in the presence of Mg^2+^. The waning of hydrophobic and polar interactions might be responsible for the denaturation of MTase with Mg^2+^; without it, MTase is very unstable and is precipitated. The binding affinity of Mg^2+^ determined using the fluorescence quenching experiment identified its strong association with MTase. The decrease in the emission intensity at 340 nm is reflected when increasing concentration of the Mg^2+^, which are accountable for the quenching of fluorescence intensity from the aromatic amino acids closely associated with Mg^2+^ (Figure 9). The ITC experiments revealed that MTase has two Mg^2+^ binding sites. The first one is stronger, and entropy plays a significant role in the binding (Table 2); the second is weaker and endothermic. Therefore, ITC experiments suggested that the MTase–Mg^2+^ complexation is mainly driven by electrostatic interaction. MST analysis was also performed to determine the Mg^2+^ binding with MTase, and these results suggested that the Mg^2+^ ion has an affinity towards MTase. 

## 4. Conclusions

Briefly, we expressed MTase and determined its activity in its shortest functional form, 33–353 amino acids of HEV polyprotein, which was previously predicted by computational studies. The active MTase was determined to be 37 kDa in size by MALDI-TOF. The activity of the enzyme was confirmed using enzymatic assay, with SAM as a methyl donor and GTP as a methyl acceptor. GDP showed the highest activity and GTP showed the second highest as a methyl acceptor in the MTase activity assay. The activity of the enzyme was found to be increased in the presence of Mg^2+^, which is a feature of many RNA capping enzymes. Similarly, the activity of MTase was decreased when it was chelated with EDTA. The circular dichroism, fluorescence quenching, and thermal denaturation studies provided the MTase with structural stability in the presence of Mg^2+^. The binding affinity of Mg^2+^ with MTase was determined by ITC and MST experiments. Overall, our study has shown the indispensable role of Mg^2+^ in MTase activity and stability. Further, this work established the optimal experimental conditions that would be helpful for the screening of inhibitor libraries against HEV MTase to identify potential inhibitors.

## 5. Materials and Methods

### 5.1. Expression and Purification of MTase

The histidine-tagged MTase (MTase) gene of approximately 960 bases coding for 33–353 amino acids of genotype 1 (GenBank accession no. AF444002.1) was cloned in pET 28a (+) vector (GenScript, New Jersey, USA) and confirmed by restriction digestion using NdeI and XhoI enzymes. The positive pET28a (+)-MTase construct was transformed into BL21 (DE3) *E. coli* cells to express the protein. A single positive clone carrying the MTase gene was grown in LB broth, and the logarithmic phase culture was induced with 1 mM IPTG at 37 °C for 3 h. The induced culture was centrifuged at 5000× *g* for 20 min at 4 °C, and the pellet was suspended in lysis buffer (10 mM Tris–Cl pH 8.0, 1 mM EDTA, 100 mM NaCl, 5 mM DTT, and 500 μg/mL lysozyme) and sonicated for 5 min (10 s on, 20 s off). The cell lysate was centrifuged for 15,000× *g* at 4 °C for 60 min. The pellet was further resuspended in 20 mL IB (inclusion bodies) wash buffer (10 mM Tris–Cl pH 8.0, 1 mM EDTA, 100 mM NaCl, 1% Triton X-100) by continuous stirring for 2 h and centrifuged at 15,000× *g* for 45 min at 4 °C. The pellet obtained in IBs was solubilised in buffer (10 mM Tris–HCl pH 8.0, 100 mM NaCl, and 0.5% NLS) (N-lauryl sarcosine, Sigma Aldrich, Burlington, MA, USA) by continuous stirring for overnight at 4 °C. The solubilised pellet was centrifuged at 15,000× *g* for 45 min at 4 °C and subsequently filtered through a 0.45 μM syringe filter (Millipore). The solubilised protein was purified using metal (Ni-NTA) affinity chromatography using AKTA start (GE Healthcare, Chicago, IL, USA). The protein was eluted in 10 mM Tris–Cl, pH 8.0, 100 mM NaCl, 0.01% NLS, and 200 mM imidazole. The purified MTase was further characterised by Western blot analysis, as described previously [33,34]. The membrane was probed with MTase epitope-specific primary antibody followed by HRP-conjugated goat anti-rabbit secondary antibody (Invitrogen, Waltham, MA, USA). Signal was detected using electrochemiluminescence (Bio-Rad Western ECL substrate, Hercules, CA, USA). 

### 5.2. Functional Aspects of MTase

The functional analysis of MTase was determined using bioluminescence-based assay with GTP as a substrate. The reaction was performed in white-bottomed 96-well plate (Tarsons) in the presence of 20 mM Tris buffer of pH 8.0, 50 mM NaCl, 1 mM EDTA, 3 mM MgCl_2_, 0.1 mg/mL BSA, 1 mM DTT, and 1 μM SAM. SAM acts as a methyl donor, and GTP acts as the methyl acceptor. In principle, MTase transfers a methyl group from SAM to GTP and converts it into SAH and m^7^GTP. Then MTase-Glo™ reagent (Promega, Madison, WI, USA) changes SAH to ADP, which is turned into ATP by MTase-Glo™ detection solution (Promega, USA). The luminescence was measured on a Tecan Plate reader. The enzymatic activity was determined under various reaction conditions, such as pH (6–12), temperature (17–45 °C), time (5–120 min), and various enzyme (0 to 5 μM) and substrate concentrations. The substrate-specificity assay was performed to test its ability to incorporate methyl groups on different nucleoside triphosphates, ATP, CTP, UTP guanine nucleotides (GMP and GTP), and cap analogues (m^7^GDP, m^7^GTP, and dGTP) at 0.5 mM. The standard reaction was carried out at 37 °C for 120 min in a 20 μL solution that contained 0.675 μM MTase, 0.4 mM GTP, 20 mM Tris buffer, pH 8.0, 50 mM NaCl, 1 mM EDTA, 3 mM MgCl_2_, 0.1 mg/mL BSA, 1 mM DTT, and 1 μM SAM. The reaction was stopped by adding 0.5% TFA (trifluoroacetic acid) (Sigma) and incubated for 10 min at room temperature, followed by incubation with MTase Glo reagent at room temperature for 30 min. Further, MTase detection reagent was added to the reaction mixture, and luminescence was detected using Tecan Plate Reader after 30 min. Similarly, the MTase activity was determined by increasing the concentrations of Mg^2+^ and EDTA. A standard curve was generated using a serial dilution of SAH ranging from 0 to 1000 nM to correlate luminescence with SAH concentration. The luminescence was plotted on *Y*-axis against the SAH concentration on *X*-axis, and the straight-line equation (Y = 19.71 × X + 227.60) was calculated using linear regression in GraphPad Prism 9.0. The graphs were plotted as a function of the concentration of SAH produced.

### 5.3. Secondary Structure Analysis Using CD Spectroscopy 

Circular dichroism (CD) spectra were performed on a Jasco J-1500 model spectropolarimeter. For the instrument calibration, (+)-10-camphor sulfonic acid was used. All CD experiments were performed at standard temperature (25 °C), which was thermostatically controlled by the Jasco Peltier PTC-423S/15 attached to the cell holder with a precision of ±0.1 °C. The change in the secondary structure of MTase under native conditions and the complex form with magnesium (Mg^2+^) ions were observed to be in the range of 200–250 nm by using a 0.1 cm cell path length. The HT voltage of the scans was kept below 600 V, and the reference signal spectrum was subtracted for each scan. The scan speed of 100 mm/min and the response time of 1 s was set for each scan measurement; each spectrum was an average of three scans. Furthermore, the secondary structure content of MTase was calculated using online DichroWeb software and Chen et al. method [24]. The spectra were smoothed by the Savitzky–Golay method with 15 convolution widths. The results were expressed as mean residual ellipticity (MRE) in deg. cm^2^ dmol^−1^, which is defined as:

(1) $$\mathrm{MRE}=\frac{\theta_{\mathrm{obs}}(m\deg)}{10\times n\times C\times l}$$

where *θ*_obs_ is the observed ellipticity in degrees, *C* is the molar fraction, *n* is the number of amino acid residues (321 − 1 = 320), and *l* is the length of the light path in centimeters.

### 5.4. Thermal Stability of MTase with Mg^2+^

A thermal denaturation study of MTase was performed on a specific wavelength (222 nm) using far-UV CD experiments, further analysed based upon the two-state unfolding model. In the case of a single-step unfolding process, N⇄U, where N refers to the native state and U to the unfolded state, and *K*_u_ for the equilibrium constant with *f*_u_ and *f*_n_ are the molar fractions of U and N, respectively.

(2) $$K_{u}=\frac{f_{u}}{f_{n}}$$

(3) $$f_{d}=\frac{\left(Y_{\mathrm{obs}}-Y_{n}\right)}{\left(Y_{u}-Y_{n}\right)}$$

where *Y*_obs_, *Y*_n_, and *Y*_u_ reflect the observed property, property of the native state, and property of the unfolded state, respectively. 

### 5.5. Fluorescence Quenching Measurements

Mg^2+^ binding with MTase was performed by fluorescence measurements and was performed on Fluorolog TCSPC Horiba FL-1057 spectrofluorometer attached to a temperature-controlled water bath with an accuracy of ±0.1 °C. The change in fluorescence intensity of MTase was observed at 340 nm and further analysed by using the Stern–Volmer equation [36]

(4) $$\frac{F_{o}}{F}=K_{sv}[Q]+1$$

where F_o_ and F are the MTase fluorescence intensities in the absence and presence of Mg^2+^ (quencher), and *K_SV_* is the Stern–Volmer quenching constant was calculated from the equation

(5) $$K_{sv}=k_{q}\cdot \tau_{o}$$

where *k*_q_ is the bimolecular rate constant of the protein–ligand reaction process and *τ_o_* is the average integral fluorescence lifetime of Trp, which is ~5.78 × 10^−9^ s [37]. Therefore, binding constants and binding stoichiometry were calculated [38,39].

(6) $$\log\left(\frac{F_{o}}{F}-1\right)=\log K_{b}+n\log[Q]$$

where *K*_b_ is the binding constant and *n* is binding stoichiometry.

### 5.6. Binding Affinity Using Isothermal Titration Calorimetry (ITC)

The binding affinity of MTase and Mg^2+^ at 25 °C was determined using isothermal titration calorimetry (ITC). MTase and Mg^2+^ were dissolved in 50 mM phosphate buffer, 50 mM NaCl, pH 7.4, and gently degassed. Forty microliters of water was added to a sample cell containing 280 µL of MTase. There was a total of 20 injections, with each infusion of 2 µL of 17 mM Mg^2+^ ion titrated into the sample cell containing 30 μM of MTase simultaneously. Intervals of 150 s separated each injection to allow the signal to return to baseline. A constant stirring speed of 750 rpm was maintained to ensure proper mixing after each infusion. Control experiments were performed under similar conditions by titrating Mg^2+^ into the buffer and were subtracted to correct for the heat of dilution. Thermodynamic parameters were obtained by fitting the data to a two-set site model using Origin software (7.0, OriginLab Corporation, Northampton, MA, USA).

### 5.7. MicroScale Thermophoresis Measurements

Further, MST experiments were conducted to find the affinity of Mg^2+^ to MTase by following the manufacturer’s protocol {Monolith NT.115 Series, NanoTemper Technologies). The process involved fluorescent labelling of MTase using a His-tag labelling kit (NanoTemper Technologies). Briefly, 50 nM of the enzyme was incubated with the labelling buffer at RT for 30 min. For Mg^2+^, the two-fold serial dilutions were made starting from 5 mM to 300 nM in 16 steps. The assay was performed in buffer (20 mM HEPES, 100 mM NaCl, 0.08% NLS, and 5% glycerol) that provides a good stability strength for protein and Mg^2+^. The mixture was loaded into glass capillaries and the MST analysis was performed using Monolith NT.115 (NanoTemper Technologies) at 25 °C. 

### 5.8. Statistical Analysis

All the statistical analyses were performed using GraphPad software, version 9.0.0 (San Diego, CA, USA). The statistical significance of the data was determined using Student’s *t*-test (unpaired).

## Figures and Tables

**Table 1:** Percentage change in secondary structure content and thermal denaturation values of MTase in the presence and absence of Mg^2+^. This revealed changes in the structural conformational and stability of MTase at pH 8.0.

Sample System	% α-Helices	% β-Sheets	% Random Coils and Other Sec. Structures	*T*_m_ (°C)
Native MTase	27.08	23.03	48.09	58.83
MTase + 0.2 mM Mg^2+^	22.64	36.96	40.40	61.57
MTase + 1.0 mM Mg^2+^	25.93	24.87	49.20	59.73

**Table 2:** Binding parameters for the MTase and metal (Mg^2+^) ion complex at standard temperature (25 °C).

Systems	*K_sv_* (M^−1^)	*K*_q_ × 10^−9^ (M^−1^S^−1^)	*N*	*K*_b_ (M^−1^)	ΔG (kcal mol^−1^)
HEV MTase–Mg^2+^	33.61± 0.02	5.81 ± 0.02	0.96	8.11 ± 0.59	−1.239 ± 0.010

**Table 3:** Thermodynamic parameters obtained from ITC thermogram.

Systems	*K*_1_ (M^−1^)	∆H_1_ (kcal/mol)	T∆S_1_ (kcal/mol)	∆G_1_ (kcal/mol)	*K*_2_ (M^−1^)	∆H_2_ (kcal/mol)	T∆S_2_ (kcal/mol)	∆G_2_ (kcal/mol)
HEV MTase–Mg^2+^	6.7 × 10^4^ ± 1.15 × 10^4^	−0.08 ± 0.06	6.49 ± 0.10	−6.57 ± 0.16	300 ± 55	4.00 ± 0.36	7.39 ± 0.44	−3.39 ± 0.09

## Data Availability

Data is available within the article and supplementary file.

## Supplementary Materials

The following supporting information can be downloaded online. Figure S1: MALDI TOF analysis of purified HEV-MTase. The major peak corresponds to the molecular weight of the HEV-MTase i.e., 37.07 kDa.

## Sample Availability

Samples of the compounds and MTase are available from the Deepak Sehgal’s laboratory for the current research work.

## Abbreviations

HEV-MTase	Hepatitis E Virus Methyltransferase
ORFs	Open reading frames
FI	Fluorescence Intensity
Mg^2+^	Magnesium ion
ITC	Isothermal Titration Calorimetry
CD	Circular Dichroism
MST	MicroScale Thermophoresis
*T*_m_	Melting point
