# Supplementary material for: Biochemical and Biophysical Characterisation of the Hepatitis E Virus Guanine-7-Methyltransferase
Source: Molecules. 2022 Feb 23;27(5):1505. doi: 10.3390/molecules27051505 (PMC8911963; doi:10.3390/molecules27051505)
Supplement: Supplementary file 1 [file molecules-27-01505-s001.zip › molecules-1553416-supplementary.pdf]

<u>**Supplementary File**</u>

Biochemical and Biophysical Characterisation of the Hepatitis E Virus Guanine-7-Methyltransferase

Preeti Hooda [1,†], Mohd Ishtikhar [1,†], Shweta Saraswat [1], Pooja Bhatia [1], Deepali Mishra [1], Aditya Trivedi [1],
Rajkumar Kulandaisamy [2], Soumya Aggarwal [3], Manoj Munde [3], Nemat Ali [4], Abdullah F. AlAsmari [4],
Mohd A. Rauf [5], Krishna K. Inampudi [2,*] and Deepak Sehgal [1,*]

MALDI-TOF

Molecular mass of the purified 37-kDa protein was determined by MALDI-TOF mass spectrometry, arbitrary index a.i.; charge-to-mass ratio, m/z. A dilution series of the peptide mixture was prepared and then mixed with a-cyano-4-hydroxycinnamic acid matrix (7.5mg/ml) in a 1;1 ratio. Each sample (0.5µl) was then spotted onto a single spot on a standard stainless steel plate in different amounts on 5800 MALDI–TOF/TOF analyzer (AB SCIEX). The laser power was set in 5500. MS spectra were acquired between 20000 to 60000m/z. and analysed by 4000 Series Explorer software, version 4.0 (AB SCIEX). The instrument was operated in positive ion mode and external calibration was performed using a calibration mixture 1 from mass calibration standard kit (AB SCIEX).

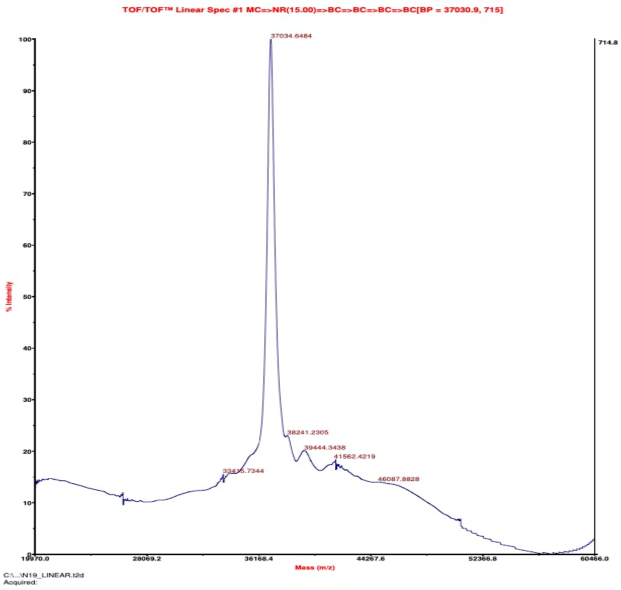

Figure S1: MALDI TOF analysis of purified HEV Methyltransferase. The major peak corresponds to the molecular weight of the HEV Methyltransferase i.e. 37.07kDa.
